# A Giant Parathyroid Adenoma Presenting as Nausea, Vomiting, and Headaches in an Adolescent Male

**DOI:** 10.1155/2023/5530269

**Published:** 2023-06-30

**Authors:** Jessica Muse, Rebecca Palmer, Jeanna Auriemma

**Affiliations:** Wake Forest Baptist Medical Center, Department of Pediatrics, 1 Medical Center Blvd, Winston-Salem, NC 27157, USA

## Abstract

Primary hyperparathyroidism is rare in the pediatric population and is typically caused by a single parathyroid adenoma. Parathyroid adenomas are almost always small and not palpable on exam but can be evaluated by neck ultrasonography or 99 m Tc-sestamibi scan. Surgical resection is the only curative treatment. In this case, a 16-year-old male presents with a 10-day history of nausea, vomiting, and headaches and is found to have a highly elevated calcium and parathyroid hormone level and a cerebral calcification in his frontal lobe noted on computed tomography. He had a palpable mass over the region of his left inferior parathyroid gland which was surgically resected with histopathology revealing a giant parathyroid adenoma. Giant parathyroid adenomas are exceptionally rare in children and adolescents and are more likely to present with severe hypercalcemic crisis than smaller adenomas. As early symptoms are often nonspecific, awareness of this clinical entity is important. There are several reports of basal ganglia calcifications in the setting of parathyroid adenoma, but, to our knowledge, this is the first report of a patient with frontal lobe calcification.

## 1. Introduction

Hypercalcemia is defined by age-specific reference ranges and in adolescence is considered a level of >10.7 mg/dL with mild, moderate, and severe concentrations being >10.7–12 mg/dL, 12–14 mg/dL, and >14 mg/dL, respectively [1]. Broadly, hypercalcemia can occur from increased bone resorption, increased gastrointestinal absorption, and decreased renal excretion. The level of parathyroid hormone (PTH) is important to differentiate between causes that are PTH-dependent and PTH-independent. PTH-dependent causes have very elevated levels of PTH and are primarily from parathyroid masses. PTH-independent causes have low PTH levels and may occur in the setting of various downstream conditions. In adults, more than 90% of hypercalcemia is from primary hyperparathyroidism (PHPT) or cancer, whereas in children, these account for less than 5% of cases of hypercalcemia [1]. More commonly in children, hypercalcemia is secondary to genetic causes such as Williams Syndrome and Down Syndrome, inborn errors of metabolism, renal disease, infection such as cytomegalovirus, chronic immobilization, vitamin D and A intoxication, and iatrogenic in premature neonates on total parenteral nutrition [1]. Causes of hypercalcemia in adolescents are more similar to adults.

## 2. Case Presentation

A 16-year-old male presents to the emergency department with a 10-day history of nausea, vomiting, and headaches. His nausea has been constant and is worse with eating and drinking. He has been having two to three episodes of nonbloody and nonbilious emesis each day. Headaches are described as tension-type, located in the bilateral temporal area. Headaches do not change with position, do not wake him from sleep, and are not improved with analgesics. Additionally, he reports polydipsia without polyuria, constipation, and several months of bone pain in his bilateral lower extremities which his family had attributed to growing pains. He had been seen several days previously in an urgent care setting and diagnosed with viral gastritis with dehydration, and antiemetics were prescribed. He comes to the emergency department because his nausea is refractory to antiemetics, and he has been unable to eat or drink without vomiting. He denies fatigue, fevers, weight loss, abdominal pain, diarrhea, muscle weakness, myalgia, arthralgia, irritability, difficulty concentrating or memory problems. He has no long-term medical problems except for obesity and has had no previous hospitalizations or surgeries.

In the emergency department, he is afebrile with a normal heart rate and respiratory rate. He is hypertensive with a blood pressure of 140/86 mmHg. On physical exam, he is nontoxic in appearance. His abdomen is soft, nontender, and without masses. His lungs are clear, and he has normal heart sounds. No goiter is present; however, a firm, palpable mass is appreciated over the inferior aspect of the left lobe of the thyroid gland. His musculoskeletal and neurological exam is normal.

Laboratory serum studies are significant for a calcium of 17.8 (8.5–10.7 mg/dL), venous ionized calcium of 2.39 (1.0–1.3 mmol/L), intact PTH level of 1,967 (12–72 pg/mL), phosphorus of 3.0 (3.0–6.0 mg/dL), 25-hydroxy vitamin D level of 10 (>30 ng/mL), and creatinine of 0.98 (0.5–1.0 mg/dL). His serum magnesium, thyroid stimulating hormone (TSH), alkaline phosphatase, and free thyroxine (T4) levels are normal. Urine studies reveal a urinary calcium to creatinine ratio of 0.06.

**Figure 1:**
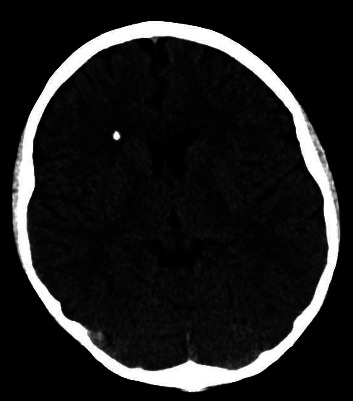
A single parenchymal calcification is noted in the right frontal lobe with no other intracranial abnormalities.

Computed tomography (CT) of his head without contrast reveals a single parenchymal calcification in the right frontal lobe with no other intracranial abnormalities (Figure 1). An ultrasound of the neck shows a hypoechoic structure measuring 1.7 cm by 3.0 cm by 3.7 cm in the area of the left inferior parathyroid gland. The thyroid gland appears normal with no nodules or masses, and there is no lymphadenopathy. An electrocardiogram (ECG) is normal including the QT interval.

He is started on aggressive intravenous saline hydration, furosemide, and vitamin D supplementation and is hospitalized for further management.

## 3. Final Diagnosis

In this patient with a markedly elevated serum calcium, a PTH more than 27 times the upper limit of normal, and a parathyroid mass on imaging, the most likely diagnosis is a PHPT secondary to a parathyroid adenoma. This case was unusual as the mass was palpable on exam which is rare for parathyroid adenomas and instead raises concern for parathyroid carcinoma due to its size. Most parathyroid adenomas are small measuring less than 2 cm and weighing less than 1 gram. Parathyroid adenomas larger than 3.5 grams are considered “giant adenomas” and are more likely to present with severe hypercalcemic crisis [2]. Upon surgical resection, our patient's parathyroid adenoma was measured at 11.2 grams; pathology was negative for malignancy.

## 4. Hospital Course

The parathyroid mass was fully resected, and due to adherence of the mass to the left thyroid lobe, the patient had a left hemithyroidectomy as well. In the operating room, PTH levels were monitored from the right internal jugular vein. The preoperative baseline was greater than 5000 pg/mL. After resection, the 5-minute level was 77.4 pg/mL, 10-minute level was 72.0 pg/mL, 15-minute level was 67.6 pg/mL, and then, a final 30-minute level was 55.9 pg/mL.

Two days postoperatively, as his PTH continued to decrease, he began to develop hypocalcemia, hypomagnesemia, and hypophosphatemia, and he was placed on continuous cardiac telemetry. Enteral electrolyte repletion was initiated with calcium carbonate, calcitriol, magnesium oxide, and potassium phosphate. Three to four days postoperatively, he exhibited signs of symptomatic hypocalcemia with emotional instability, fatigue, and perioral and acral paresthesia at a calcium nadir of 6.9 mg/dL. His symptoms resolved with intravenous calcium gluconate. His electrolytes stabilized, and he was discharged home six days postoperatively on calcium carbonate, calcitriol, Vitamin D3 (cholecalciferol), and levothyroxine. Levothyroxine was started empirically given his hemithyroidectomy.

Genetic testing was performed including genes associated with multiple endocrine neoplasia (MEN) and hyperparathyroidism-jaw tumor syndromes (*MEN1*, *RET*, *CDKN1B*, and *CDC73*) which were all negative.

## 5. Discussion

The parathyroid gland is essential for maintaining serum calcium levels. PTH responds to serum calcium levels via calcium-sensing receptors (CaSR) on the plasma membrane of parathyroid cells and in turn acts at both the skeletal and renal system to maintain calcium levels. PTH activates osteoclasts to resorb bone and release calcium. In the kidney, PTH increases renal reabsorption of calcium and stimulates the conversion of 25-hydroxy vitamin D to the active metabolite calcitriol (1, 25 di-hydroxy vitamin D). Calcitriol assists PTH in osteoclast activity, renal reabsorption of calcium and also increases the intestinal absorption of calcium [3]. In primary hyperparathyroidism (PHPT), there is excessive secretion of PTH from one or more of the four parathyroid glands with subsequent hypercalcemia [4].

PHPT is rare in the pediatric population affecting only 2–5 in 100,000 children and adolescents [4]. In contrast, the incidence in adults is 66 in 100,000 women and 25 in 100,000 men [5]. PHPT results from a single parathyroid adenoma in 90% of cases [4]. Giant parathyroid adenomas as seen in our patient are exceptionally rare in the pediatric literature with only a few cases reported [6]. MEN-1 is the most common cause of inherited PHPT [4]. Nearly 100% of individuals with MEN-1 will have primary hyperparathyroidism by age 50 [7]. Men-2A is characterized by increased risk for medullary thyroid carcinoma, primary hyperparathyroidism, and pheochromocytomas [7]. MEN-4 is a newer entity, first described in 2006. These patients develop MEN-1-associated tumors without a mutation in the MEN-1 gene. A mutation in the *CDK1B* has been identified in many of these individuals [8]. Our patient did not have a family history of parathyroid adenomas, neuroendocrine tumors, adrenal tumors, or thyroid adenomas. PHPT may also be associated with hyperparathyroidism-jaw tumor syndrome which is associated with mutations in the *CDC73* gene and may be accompanied by ossifying fibromas of the jaw [9].

PHPT should be differentiated from familial benign hypocalciuric hypercalcemia (FBHH) as the latter does not cause end-organ damage and warrants no treatment. Parathyroidectomy does not normalize calcium levels in FBHH. FBHH is much more common than PHPT, with a prevalence of 1 in 16,000, and is typically caused by loss of function mutations in the CaSR gene [10]. PHPT and FBHH can be differentiated based on lab values: although both cause hypercalcemia, FBHH typically has a normal to only mildly elevated PTH level and will have a low calcium to creatinine ratio of 0.001–0.018 [10]. Our patient's calcium to creatinine ratio of 0.06 would make FBHH very unlikely, even before imaging showed a parathyroid mass.

Many adults with PHPT are diagnosed incidentally when routine laboratory studies reveal hypercalcemia; however, given children and adolescents less commonly have routine laboratory studies performed, and most children and adolescents present with late findings of symptomatic hypercalcemia and end-organ damage [3, 4]. Clinical symptoms and findings seen in children with PHPT may include nephrolithiasis, nephrocalcinosis, bone pain, myalgia, arthralgia, abdominal pain, nausea, and vomiting (secondary to a hyper-acidic gastric environment, constipation, or pancreatitis), polyuria, polydipsia, and neurocognitive symptoms (including irritability, difficulty concentrating, and memory disturbances). The phrase “stones, bones, abdominal groans, thrones, and psychiatric overtones” is a helpful way of remembering these features of PHPT; however, children and adolescents may present with a variety of nonspecific complaints [4]. Initial management of hypercalcemia while initiating a work-up for cause includes aggressive saline hydration and use of loop diuretics such as furosemide to increase renal calcium excretion.

In patients with suspected PHPT, neck ultrasonography or 99 m Tc-sestamibi scan is useful to identify a parathyroid adenoma preoperatively and allow for targeted minimally invasive surgery [4]. Surgical removal of the adenoma is the only definitive treatment, and intraoperative monitoring of PTH levels is performed to determine if resection is successful. A postsurgical drop of more than 50% is considered curative [11].

Complications of parathyroidectomy include laryngeal nerve injury, recurrence of illness, postoperative hypocalcemia, and prolonged, severe hypocalcemia often termed “hungry bone syndrome” [12, 13]. Hungry bone syndrome is defined as hypocalcemia <8.4 mg/dL and lasting greater than 4 days postoperatively. This phenomenon is thought to be from decreased osteoclast activity in response to normalized PTH with bone remineralization [13]. Hypophosphatemia, hypomagnesemia, and hyperkalemia may also be seen. Frequent electrolyte monitoring and cardiac telemetry is crucial after parathyroid gland removal. Patients with significant postoperative hypocalcemia may develop paresthesia (most commonly perioral and acral, as seen in our patient), emotional instability, carpopedal spasms, seizures, tetany, and laryngospasm [4]. Latent tetany may be appreciated by the Chvostek and Trousseau sign [4]. However, postoperative hypocalcemia may be asymptomatic. An ECG may show a prolonged QT interval which can precipitate ventricular arrhythmias if severe hypocalcemia occurs [4]. Symptomatic hypocalcemia or ECG changes should prompt urgent intravenous calcium replacement.

The incidental finding of a benign solitary cerebral calcification on CT imaging of our patient is interesting due to its location in the frontal lobe. Cerebral calcifications associated with parathyroid adenoma were first described in 1956, and at least 2 additional cases have been reported; however, in all previous cases, calcification was located in the region of the basal ganglia [14, 15]. To our knowledge, this is the first report of frontal lobe calcification associated with parathyroid adenoma.

## 6. Lessons for the Clinician

- Hypercalcemia may present with non-specific complaints including nausea, vomiting, and headaches
- Primary hyperparathyroidism is almost always secondary to a parathyroid adenoma in children and adolescents, and these are rarely palpable on exam
- MEN-1 syndrome is the most common cause of inherited PHPT
- The postoperative course of parathyroidectomy is complicated by the need for vigilant electrolyte monitoring and may include severe, prolonged hypocalcemia due to “hungry bone syndrome”

## Data Availability

No underlying data were collected or produced in this study
